# Exploring the Cardiovascular Impacts of Agmatine: A Systematic Review

**DOI:** 10.3390/medsci13040255

**Published:** 2025-10-31

**Authors:** Oana-Mădălina Manole, Gabriela Rusu-Zota, Amin Bazyani, Viviana Onofrei

**Affiliations:** 1 Internal Medicine Department, Faculty of Medicine, “Grigore T. Popa” University of Medicine and Pharmacy, 700115 Iasi, Romania; 2 Cardiology Department, “Saint Spiridon” Emergency Clinical County Hospital, 700115 Iasi, Romania; 3 Pharmacology Department, Faculty of Medicine, “Grigore T. Popa” University of Medicine and Pharmacy, 700115 Iasi, Romania; 4 Pharmacology Department, “Saint Spiridon” Emergency Clinical County Hospital, 700115 Iasi, Romania; 5 Interventional Department, Cardiovascular Diseases Institute “Prof. Dr. George I. M. Georgescu”, 700115 Iasi, Romania

**Keywords:** imidazoline agonist, agmatine, cardiovascular system

## Abstract

Background: Agmatine (AG) is an endogenous neurotransmitter discovered in 1910. It acts on imidazoline I1 and I2 receptors, alpha-2 adrenoceptors, N-methyl-D-aspartate receptors (NMDAR), and serotonergic receptors and modulates nitric oxide synthase (NOS) subtypes. It has neuroprotective, anxiolytic, antidepressant, anticonvulsant, and anti-inflammatory properties and is involved in cognitive functions and withdrawal. The cardiovascular effects of AG began to be explored after the hypotensive effect of clonidine, an imidazoline agonist, was demonstrated. The current study aimed to systematize the effects of AG on the cardiovascular system obtained in previous preclinical studies. Methods: We searched three databases, PubMed, Cochrane, and Embase, using the keywords “agmatine” and “cardiac” or “vascular.” Results: Sixty studies were eligible and included in the analysis. Initially identified as Clonidine Displacing Substance (CDS), AG has demonstrated dual effects—an increase or decrease in blood pressure or in heart rate. Conclusions: The effects exerted by AG depend on the dose and route of administration, as well as on the receptors involved and the pathophysiological pathway used.

## 1. Introduction

**Figure 1:**
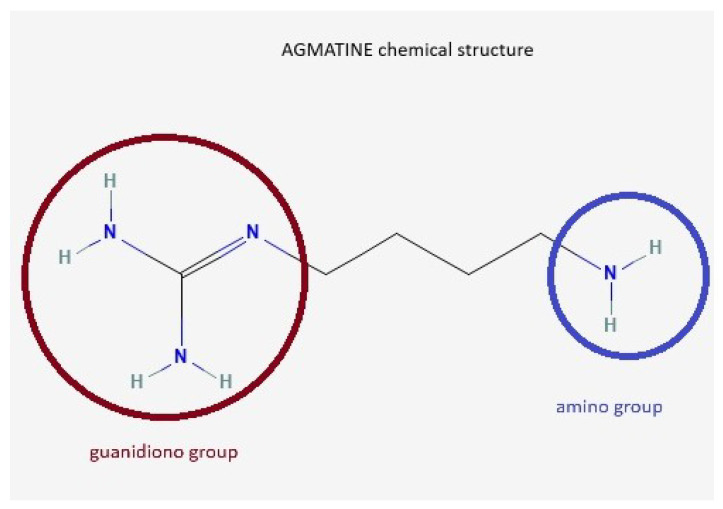
AG chemical structure. AG, known as 4-aminobutyl-guanidine, has a guanidino group (red circle) and an amino group (blue circle), which enables it to act as a divalent ion at physiological pH.

Agmatine (AG) is an endogenous neurotransmitter identified by Albrecht Kossel in 1910 [1], involved in cognitive functions, learning, and memory [2]. It is synthesized from L-arginine under the action of the enzyme arginine decarboxylase [3,4,5]. Other arginine precursor compounds are nitric oxide (NO), citrulline, and ornithine [6]. The chemical structure of AG contains a guanidino group and an amino group (Figure 1).

**Figure 2:**
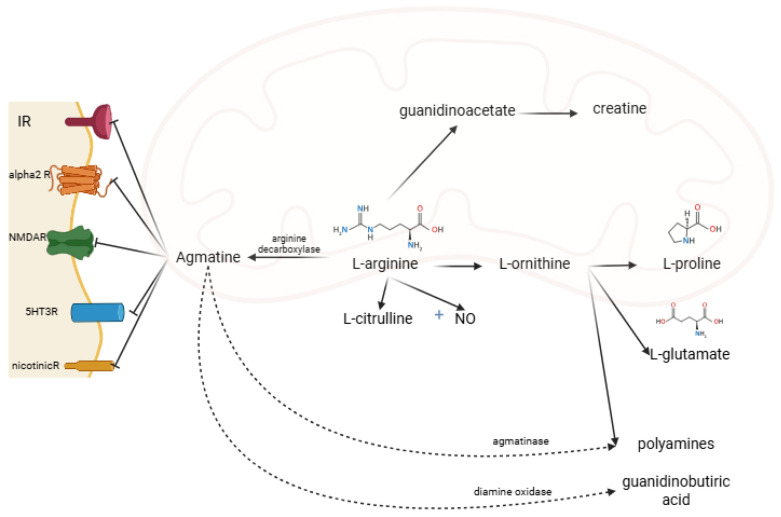
AG metabolism, and receptor binding sites. IR, imidazoline receptors; alpha2R, alpha-2 adrenergic receptors; NMDAR, N-methyl-D-aspartate receptors; 5HT3R, 5-hydroxytryptamine type 3 receptor; nicotinicR, nicotinic receptors; NO, nitric oxide (created in BioRender. Manole, O-M (2025) https://app.biorender.com/illustrations/68191cdac647b23d23575abb, accessed on 8 August 2025).

Under the action of diamine oxidase, the guanidino group is metabolized to gamma-aminobutyric acid. The amino group is metabolized by agmatinase and agmatine-like protein to urea and polyamine (putrescine, subsequently spermine and spermidine) [4,5]. AG acts on several receptors and enzymes. It is an agonist on imidazoline (I1, I2) and alpha-2-adrenergic and an antagonist on N-methyl-D-aspartate receptors (NMDAR) [2,7] (Figure 2). AG acts differently on the isoenzymes of nitric oxide synthase (NOS): it inhibits the neuronal NOS subtype (nNOS) and inducible NOS (iNOS) subtype and stimulates the endothelial NOS (eNOS) subtype [8].

**Figure 3:**
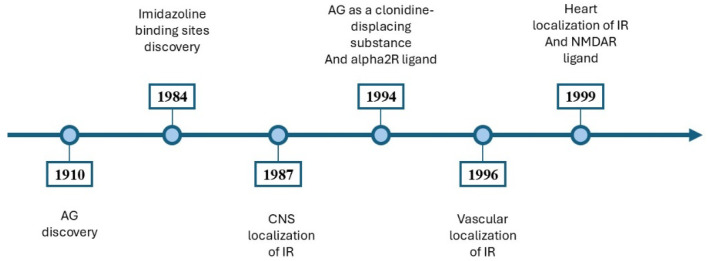
An overview of the main discoveries involving AG. In 1910, Albrecht Kossel isolated the AG in herring sperm [1]. In 1984, Bousquet et al. [15] introduced the concept of imidazoline binding sites [15]. In 1987, Ernsberger et al. [16] showed the CNS localization of imidazoline binding sites [16]. In 1994, AG was proposed as a clonidine-displacing substance and an alpha2R ligand [17]. In 1996, imidazoline binding sites were identified on vascular smooth muscle and endothelium [18]. In 1999, imidazoline binding sites were identified in cardiovascular tissues [19], and AG is a ligand for NMDARs [20]. AG, agmatine; CNS, central nervous system; IR, imidazoline receptors; NMDAR, N-methyl-D-aspartate receptors; alpha2R, alpha-2 adrenergic receptors.

The neuroprotective, antidepressant, and anticonvulsant effects of AG have been demonstrated [2,3,7,9,10,11]. AG had anti-inflammatory effects through modulating the prooxidative and antioxidative balance in the hippocampus [3]. It inhibited the addiction process [12,13] and attenuated the symptoms of ethanolic, nicotinic, and morphine withdrawal [12,13,14]. A timeline outlining the significant milestones in the study of AG is illustrated in Figure 3.

Because of its properties, AG is considered a neurotransmitter; it is distributed throughout the body, reaching concentrations of 2.4 ng/g in the brains of rats, 6.3 ng/g in the hearts of rats, and 57.41 ng/g in the aortas of rats [21].

The effects of AG on the cardiovascular system have drawn attention since 1994, when Li et al. [17] identified AG using mass spectroscopy as a “clonidine-displacing substance” (CDS) in bovine brain [17]. Clonidine, a well-known first-generation central antihypertensive, decreased sympathetic tone through agonist action on I1R in the rostral ventrolateral medulla (RVLM). Due to its adverse effects (such as xerostomia, sedation, and depression), researchers continued to investigate other central agents [22]. Moxonidine and rilmenidine, second-generation central antihypertensives with a greater selectivity for IR rather than alpha-2R, act similarly but have fewer side effects. The discovery of AG as a potential CDS, with agonistic action on IR and alpha-2R, had focused research on its potential cardiovascular effects [23]. However, other authors obtained contradictory results [24]. AG plays an important role in cardiovascular function [25,26,27], and the mechanism of action is poorly understood.

The article aims to provide a systematic review of the effects of AG on the cardiovascular system and its mechanisms.

## 2. Materials and Methods

### 2.1. Search Strategy and Registration

In order to identify studies that evaluated the cardiovascular properties of AG, a search was conducted in databases such as PubMed, Cochrane Library, and Embase on 15 June 2025. The PRISMA statement was followed to conduct the present systematic review [28]. The search strategy combined MESH terms using the Boolean operators “AND” and “OR”. The terms used in the search were “agmatine”, “cardiovascular”, and “vascular”. A supplementary search was carried out in the references of the studies included.

### 2.2. Eligibility Criteria

Both title and abstract, as well as full texts, were reviewed to establish the inclusion of a study. Because AG is primarily explored in experimental medicine, the majority of the research was preclinical studies, and the inclusion criteria were adjusted accordingly as follows: (1) original reports, (2) contained at least one experiment assessing a cardiovascular measure, (3) used AG in a control, comparator, or experimental context, (4) full-text articles, (5) English language, and (6) published before June, 2025.

**Figure 4:**
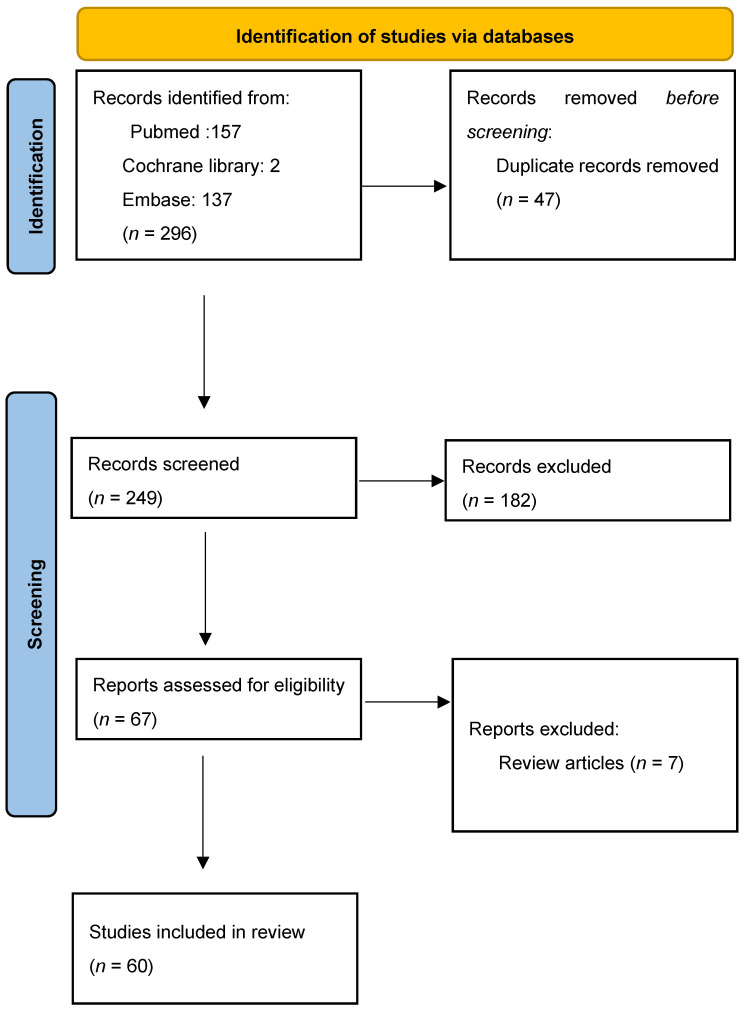
PRISMA flow chart for study design.

A total of 296 records from all years were generated. Firstly, we found 47 duplicates and removed them. Of the 249 screened results, 182 records were excluded. Exclusion criteria were that the article was not suitable (*n* = 168), the full text was not in English (*n* = 8), or it was an editorial (*n* = 6). A total of 67 full-text articles were assessed for eligibility. After excluding review articles (*n* = 7), a total of 60 articles were included in the systematic review. Table S1 summarizes the information from the selected articles. The PRISMA flow chart is illustrated in Figure 4.

### 2.3. Quality Assessment and Risk of Bias

To ensure the highest quality, two researchers independently reviewed and assessed each extracted study according to the PRISMA guidelines [28]. A PRISMA checklist is provided in the Supplementary Materials.

The risk of bias was evaluated using SYRCLE’s Risk of Bias (RoB) tool, a version of the Cochrane RoB tool adapted for assessing laboratory animal studies. This tool covers 10 criteria addressing selection, performance, detection, attrition, reporting, and other potential sources of bias [29]. The overall risk of bias of all studies was assessed with robvis tool [30].

The data extracted from the studies included in this systematic review were summarized in Table S1 (Supplementary Material), giving the following information: author and year of publication, animal model used, the observed effects, and underlying mechanisms. The assessment of the total risk of error revealed some concerns. Studies published before the standardization of preclinical research methodology showed an increased number of selection and performance errors, as well as uncertainty in the accuracy of data reporting. This review also included studies conducted on tissues. In these cases, the sequence of allocation of compared groups or accommodation conditions was not reported.

## 3. Cardiovascular Effects of AG

### 3.1. The “Clonidine-Displacing Substance”-like Cardiovascular Activity of AG

The history of the discovery of IR is closely linked to the identification of clonidine [31]. Clonidine is an aminoimidazoline developed by Stähle in the 1960s. Aminoimidazolines are chemical compounds containing a nitrogen atom between an imidazoline ring and a phenyl ring. The term “imidazoline” comes from the chemical structure characteristic of clonidine. Clonidine was the first centrally acting antihypertensive proposed compound. It is an agonist of IR and alpha-2R [31].

In 1984, Atlas and Burstein revealed the existence of CDS in a purified bovine brain extract [32]. A decade later, Li and collaborators [17] identified AG through mass spectrometry as a CDS in bovine brain tissues [17]. Since then, the cardiovascular effects of AG have been a subject of interest. It has been tested across various animal models and even in humans, using different routes of administration and a range of dosages.

As a proposed CDS, intravenous (iv) bolus administration of AG decreased systemic arterial pressure and systemic vascular resistance in a concentration-dependent manner, with a maximum effect at a dose of 10 mg in anesthetized Sprague Dawley rats in vivo. This effect showed tachypylaxis but did not exhibit cross-tachypylaxis with agents such as nitroglycerin, bradykinin, or isoproterenol [33].

Moxonidine and rilmenidine are second-generation central antihypertensives with a greater selectivity for IR over alpha-adrenergic receptors. To assess whether their antihypertensive effects were mediated predominantly through IR rather than adrenergic receptors, Head [34] administered these drugs intracisternally at clinically relevant doses to conscious rabbits. All three compounds lowered blood pressure (BP); rilmenidine was eleven times weaker than clonidine, while moxonidine was three times more effective. The hypotensive effects of moxonidine and rilmenidine were mainly attributed to I1R activity, while clonidine’s effect involved both I1R and alpha2-adrenergic receptors. The administration of low doses of AG (0.01–10 μg/kg) caused minimal changes in BP, but led to moderate bradycardia (a decrease of 16 ± 3 bpm) in contrast to higher doses (100 μg/kg), which increased BP by 22 ± 4 mmHg and counteracted the bradycardia. Combining AG with either clonidine or moxonidine did not influence their hypotensive effects, but intensified the bradycardia. When AG was paired with imidazoline and alpha-2-adrenergic antagonists, such as efaroxan and metilidazoxan, the bradycardia was reversed [34,35]. Therefore, these findings suggest that AG can imitate clonidine’s bradycardic effects, but it lacks significant hypotensive action as a CDS.

Meanwhile, another group of scientists reported similar findings. When AG was administered intracisternally (directly into the cisterna magna) to conscious rabbits at doses of 30, 100, and 300 μg/kg, the BP, the renal sympathetic nerve activity, and the plasma concentration of NA and adrenaline increased [36]. Although there was a slight but statistically insignificant decrease in heart rate (HR), the sympathoexcitatory effects of AG were attributed to the involvement of IR [36].

Further research indicated that AG did not replicate the cardiovascular effects of clonidine. When administered intracisternally, AG (100 and 400 nmol) exhibited a dose-dependent vasopressor activity and increased sympathetic nerve activity, while iv administration produced a dose-dependent vasodepressor activity in anesthetized ventilated rats. The microinjection of AG into the RVL failed to lower BP, and iontophoretic application did not reduce the firing of RVL-spinal vasomotor neurons, supporting that AG’s action is not attributed to direct action on neurons in the RVL. When administered systemically (60 μmol iv), AG reduced BP by inhibiting postganglionic sympathetic nerve output and exerting a direct action on vascular smooth muscle [37].

When administered either into the renal artery (at doses of 0, 3, 10, 30, or 100 nmol/kg/min in a 3.4 μL/min solution) or intracerebroventricularly (icv) (at doses of 0, 10, 100, 300, or 1000 nmol in 5 μL) in Sprague Dawley rats, AG did not produce any detectable changes in BP or HR [38].

To further explore the effects of AG as a CDS, Schaffer and collaborators [39] evaluated its administration via both peripheral and central routes. When AG was administered into the fourth ventricle, it caused an increase in HR, while iv administration decreased the HR dose-dependently in anesthetized more than pithed spontaneous hypertensive rats (SHRs). The decrease in HR was potentiated when AG was administered through iv alongside a long-term clonidine infusion. Conversely, the icv administration of AG did not exert any effect on HR. The increase in HR following the fourth ventricle administration may involve the nucleus tractus solitarius. Systemic effects are believed to result from the inhibition of sympathetic ganglionic transmission [39]. Similar results were reported by Raasch and collaborators [40] and Briaud and collaborators [24]. The iv administration of AG decreased HR more significantly in anesthetized than in pithed SHRs. Administration into the fourth ventricle increased HR in anesthetized SHRs. In pithed SHRs, a pretreatment with AG enhanced the minimal HR-increasing effect of clonidine, while, in anesthetized rats, it amplified the HR decrease. AG may modulate NA release via imidazoline binding sites. This involves a decrease in sympathetic tone and, consequently, a decreased HR [40]. Briaud and collaborators [24] suggested that the modest HR increase observed after administering low doses of AG (30–100 g/kg) into the fourth ventricle of conscious SHRs could be due to AG blockade of the glutaminergic facilitatory interneurons that activate the vagus cranial nerve [24].

Schaffer and collaborators [39] showed that AG decreased BP whether administered peripherally by iv or centrally into the fourth ventricle, in both pithed and anesthetized SHRs. However, when administered through icv, AG increased BP. An in vitro administration in the thoracic aorta showed no significant effect. Systemic effects of AG are thought to result from the inhibition of sympathetic ganglionic transmission, while the increase in BP following icv administration is likely due to the activation of pressor centers within RVLM via forebrain projections [39]. Similarly, Raasch and collaborators [40] demonstrated that AG did not fully replicate CDS-like cardiovascular activity. Iv AG administration in anesthetized SHRs caused a drop in BP, but AG did not antagonize the effect of clonidine in pithed or anesthetized SHRs. A central administration into the lateral ventricle led to increased BP in anesthetized SHRs. AG seems to modulate NA release via IR, reducing sympathetic tone in a way similar to clonidine. Although both AG and clonidine decreased BP in anesthetized SHRs, AG did not antagonize clonidine’s BP, suggesting that AG does not act as a CDS-like agent [40]. In conclusion, the initial properties of CDS cannot be attributed to AG, but other substances must be identified as CDS. The effects of agmatine varied depending on the route of administration, suggesting the involvement of multiple mechanisms of action. Table 1 summarizes the effects of the central and peripheral administration of AG.

### 3.2. The Vascular Effects of AG

Following the evidence that AG influences BP through different mechanisms from those of clonidine, subsequent research began to focus on the vasodepressor properties of AG.

Initially, AG’s ability to reverse the action of carbachol and phenylephrine on the rat aorta with an intact endothelium was tested. The AG did not reverse the responses in aortas pretreated with these compounds. However, in endothelium-denuded aortas previously exposed to endotoxin, 300 μM of AG restored contractile responses by inhibiting iNOS activity [41].

In 1996, researchers showed that AG inhibited the activity of all three isoforms of NOS [42].

In the same year, Gonzales and collaborators [43] demonstrated that AG can modulate the contraction of blood vessels. In rat tail arteries, AG (0.03–1 nM) inhibited contractions induced by transmural nerve stimulation through two mechanisms: first, by acting as an agonist at prejunctional alpha2R to suppress neurally mediated vasoconstriction, and second, by causing a delayed prejunctional facilitation of sympathetically mediated vasoconstriction [43]. Additionally, another study showed that the administration of AG decreased BP in rats and rabbits and induced vasodilation in isolated aortic rings, regardless of the presence of endothelium and independent of NO or cGMP pathways. The vasodilatory effect in aortic rings was enhanced by moxonidine, indicating the involvement of IR [44].

AG in doses of 0.1, 0.5, and 1 mg/kg exhibited varying effects on vascular tone depending on the vascular bed from Sprague Dawley rats. When injected locally, it caused dose-dependent vasoconstriction in the femoral and renal arteries, primarily through alpha-2R. In contrast, a local administration in the mesenteric artery resulted in vasodilation, an effect mainly mediated by IR [45]. Molderings and collaborators [46] showed that AG exhibited a dual interaction with the rat 2D-adrenoceptor: it is a positive modulator at an allosteric site of 2-adrenoceptors and an antagonist at the ligand recognition site of the 2-adrenoceptor [46].

Zhao and Ren [47] found that AG lacked vasoconstrictive effects on the thoracic aorta or ear vein of rabbits, in contrast to moxonidine and clonidine, which elicited vasoconstriction via alpha2R [47]. Additionally, AG (0.3–1000 mM) induced concentration-dependent relaxation in endothelium-intact aortic rings from rats that had been precontracted with phenylephrine. This relaxation effect was diminished when the endothelium was removed and inhibited by the NOS inhibitor L-NIO, but remained unaffected by AGN, an I1R antagonist. The findings suggest that AG stimulates NOS primarily through an I1R-independent mechanism [48]. In subsequent research, Zhao and collaborators [49] evaluated the effects of AG on vasoconstriction produced through electrical stimulation in the isolated rabbit saphenous artery. At a concentration of 0.1 to 1 nM, AG produced a biphasic effect: it initially inhibited the purinergic vasoconstriction, then, after 20–25 min, facilitated vasoconstriction. These effects appeared to be independent of prejunctional adrenoceptors and were likely mediated via IR [49].

Other studies have examined the vascular action of AG on both the thoracic aorta [50] and mesenteric arterioles isolated from Sprague Dawley [50,51] and Dahl salt-sensitive rats [51]. In these studies, the thoracic aortas from male Sprague Dawley rats were placed in a cold modified Krebs bicarbonate solution. Phenylephrine, at concentrations of 10^−7^ M and 3 × 10^−8^ M, caused contractions in aortas with intact and denuded endothelium, respectively. Application of 10^−10^ to 10^−3^ M of AG induced a concentration-dependent relaxation with 82% +/–5% of the precontracted, endothelium-intact thoracic aorta. The vasodilatory effect of AG in rat aortas was mediated by the activation of protein kinase B/Akt to phosphorylate eNOS and increase cyclic GMP levels, with the activation of small conductance Ca2+-activated K+ channels and ATP-sensitive and inward rectifying K+ channels [50]. Furthermore, L-arginine, a precursor to AG, also induced relaxation in isolated mesenteric arterioles from male Sprague Dawley rats (with an EC_50_ of 5.8 ± 0.7 mM), an effect blocked by the pre-administration of the endothelial arginine decarboxylase inhibitor, DFMA, indicating the involvement of AG in this response. The direct administration of AG caused vessel relaxation at concentrations 100 times lower than those of L-arginine (EC_50_ 138.7 ± 12.1 μM) [51]. In Dahl salt-sensitive rats, an AG-mediated relaxation of mesenteric arterioles was also observed, with the degree of relaxation differing based on salt intake (90.4 ± 1.7% with normal-salt and 19.8 ± 2.4% with high-salt diets) [51].

The role of IR in modulating vascular function was studied in SHRs. Iv AG administration in doses below the doses needed to cross the blood–brain barrier (1, 3, or 5 mg/kg) decreased SBP dose-dependently in SHRs, but not in Wistar–Kyoto rats. The effect was blocked by BU-224, suggesting the implication of peripheric I2R. Also, the expression of IR was observed to be higher in SHRs. Tested in isolated aortic rings from SHRs, AG induces a relaxation mediated by I1R, PKA, and KATP [52].

Similar results were obtained in another study. AG (10 μM) induced relaxation of the aortic rings from rats precontracted by phenylephrine or KCl. The effect was attenuated by BU224 (antagonist of I2) and glibenclamide (ATP-sensitive potassium channel blocker), suggesting that the effect was caused by opening ATP-sensitive potassium channels (KATP) via peripheral imidazoline I2R [53].

AG acts on different receptors, such as IR and prejunctional adrenoceptors or NOS isoforms, and exhibits a dual effect—vasodilatation and vasoconstriction—depending on the substrate, dosage, and route of administration.

### 3.3. Hemodynamic and Vascular Actions of AG

An administration of 30 and 60 µM/0.1 mL of AG solution led to a sustained decrease in BP, which lasted approximately 10 min. When AG was given after several hours or following four weeks of coadministration with L-NAME, a NOS inhibitor, the drop in BP was more pronounced and persisted for several hours. AG also reversed phenylephrine-induced contractions and attenuated the relaxant response to acetylcholine in rat thoracic aorta samples. These observations suggest a negative feedback mechanism between the two arginine metabolic pathways—arginine decarboxylase and NOS. The amplified hypotensive response may involve an enhanced baroreceptor reflex activity through the modulation of sympathetic outflow [54].

When 5 nmol of AG was administered into the RVLM of anesthetized rats, it produced a decrease in HR (approximately 20 beats per minute) and a reduction in BP (around 10 mmHg). While the effect on HR was comparable to that of clonidine, the BP response was about half. The actions of both AG and clonidine were abolished by a prior administration of idazoxan (IDZ). The inconsistent BP findings may be attributed to the relatively weak effect of AG, the specific sensitivity of the RVLM site, and the influence of the anesthesia [55]. Additionally, AG blocked the baroreflex response activated by an electrical stimulation of the left aortic nerve in anesthetized rats and abolished the pressor action of L-glutamate administration, an effect similar to that seen with clonidine [55]. Qin and He [56] also demonstrated that a perfusion of 5 mmol/L of AG inhibited carotid baroreflex in Sprague Dawley rats through IR and α2R [56].

In conscious SHR, AG exhibited dose-dependent effects. When administered into the fourth ventricle at low doses of (10–100 μg/kg), AG had no significant impact on BP. However, at higher doses of 1000 μg/kg, it produced a marked and sustained increase in BP, accompanied by convulsive reactions. Similarly, the intrascisternal administration of lower doses of AG failed to reverse the BP-lowering effect of clonidine, but higher doses were able to counteract the antihypertensive action of rilmenidine and clonidine 80 min after injection. It is well established that the bradycardic and hypotensive effects of clonidine involve α2R. However, the reversal of clonidine’s bradycardic effect by AG appears to engage receptors other than α2. Furthermore, the neurotoxic effects observed with higher doses of AG in conscious SHRs may be linked to hypoxic conditions and glutamate accumulation [24].

AG was shown to decrease the total peripheral resistance index and mean arterial BP by promoting vasodilatation in both anesthetized Dahl salt-sensitive rats (DS), which develop hypertension on a high-sodium diet, and in normotensive Dahl salt-resistant rats (DR) [57]. AG was involved in peripheral sympatho-inhibition within the systemic vasculature, leading to a reduced diastolic BP in pithed male Wistar normotensive rats [58].

Furthermore, the administration of AG (1, 10, or 20 mg/kg iv) resulted in dose-dependent decreases in left ventricular BP, cardiac index, and LP dp/dt in both anesthetized DS and DR rats. These effects were primarily attributed to a diminished cardiac output, which stemmed from both the decreased contractility and AG-induced vasodilation [57].

### 3.4. Electrophysiological Actions of AG

The influence of AG was further evaluated on sinoatrial cells from the right atrium (in doses of 1, 5, 10, and 15 mmol/L) and atrioventricular node cells (in doses of 5, 10, and 15 mmol/L) of rabbits. AG, in a dose-dependent manner, decreased the pacemaker firing rate, the amplitude of action potentials, the maximal rate of depolarization, and the velocity of diastolic depolarization, while prolonging the 90% duration of the action potential. These effects appear to be mediated by IR and alpha2R through a reduction in calcium influx and potassium efflux [59,60]. Similar mechanisms were also demonstrated when AG was administered to guinea pigs to address early and delayed afterdepolarization induced by isoproterenol in papillary muscles. AG decreased both EAD and DAD, and this effect was abolished by IDZ, but not by L-NAME, suggesting that the process is mediated by an IR- and alpha2R-dependent calcium influx [61]. However, in a study conducted by Zhao and Ren [47], AG dose-dependently reduced the pacemaker firing rate, and APD in rabbit sinoatrial cells was not affected by yohimbine, indicating that alpha-2R was not involved [47]. Contrasting previous findings, AG failed to alter the inotropy of left atrial tissue from male Wistar rats in doses of 1 nM–100 microM [62]. The contrasting results of this study may be due to the low doses of agmatine used.

AG was found to increase the contraction force in frog myocardium following transient stimulation. This positive inotropic effect was abolished by yohimbine, indicating that AG facilitates sympathetic neurotransmission via prejunctional alpha-2R on sympathetic nerve terminals [63].

AG exerted a stronger inotropic and chronotropic effect than clonidine on isolated rat atrial tissue. These actions were blocked by IDZ and phentolamine, suggesting that both IR and alpha2-/alpha-1-R are involved in the response [64].

Cobos-Puc and collaborators demonstrated that AG, administered at doses of 1000 and 3000 μg/kg/min, exerted a sympatho-inhibitory effect during electrically induced sympathetic stimulation or iv infusion of exogenous NA in pithed male Wistar normotensive rats [65] and SHRs [66]. At lower doses, the inhibitory action is mediated primarily through prejunctional alpha2R, whereas higher doses involve additional postjunctional blockades and I1R [65,66]. One year later, it was shown that AG infusion in a dose of 1000 μg/kg/min inhibited the electrically induced tachycardic responses at all frequencies of stimulation, with the implication of alpha-2R subtypes (alpha2A-R mainly, but also alpha2C-R) and I1R [67]. AG further suppresses peripheral sympathetic tone by inhibiting N-type calcium channels via the activation of I2R [68]. Its role in modulating vasodepressor sensory calcitonin gene-related peptide (CGRP)-ergic outflow was tested in pithed male Wistar rats, where the administration of 3 µg/kg/min moxonidine and 3000 µg/kg/min AG inhibited CGRP-mediated vasodepressor outflow. This effect was found to be predominantly mediated via I1R [69].

Nakipova and collaborators investigated the mechanisms regulating SR activity in hibernating animals. The research analyzed the rhytmo-inotropic effect of one-hour exposure to 500 μM AG on the right ventricle papillary muscle of Spermophilus undulatus (ground squirrels) after one hour of exposure to 500 μM. AG increased the contraction force at low stimulation frequencies (0.03–0.3 Hz), an effect likely mediated by the activation of store-operated Ca-channels (SOC channels) [70].

A research group tested the effect of AG (5 mM and 10 mM) on human atrial fibers from the apex of the right atrium appendage. AG decreased the automaticity and transmembrane potential by blocking calcium influx via IR or alpha2R. It decreased the velocity of diastolic depolarization, thus decreasing the rate of pacemaker firing. Also, it decreased the action potential amplitude, maximum upstroke velocity of phase 0 depolarization, and action potential duration at 50 and 90% of repolarization in a concentration-dependent manner [71].

AG influences the sinoatrial cells from the right atrium and atrioventricular node cells via IR and alpha-2R by modulating calcium homeostasis.

### 3.5. The Cardioprotective Effects of AG

AG has demonstrated anti-ischemic properties in a model of ischemic heart injury. AG administered intraperitoneally (ip) at a dose of 100 mg/kg body weight before the experiment reduced cellular injury in the isolated ischemic-reperfused hearts and improved coronary flow and mechanical left ventricular performance in male Wistar rats [72]. Two years later, the researchers explored the dose–response effect and the possible mechanism involved in the protective effect. It was shown that low doses of AG (100 μm/L) given ip pre- and post-ischemia and high doses of AG given ip pre-ischemia significantly improved the mechanical left ventricular performance and myocardial recovery of ischemic-reperfused isolated rat hearts due to the inhibition of poly (ADP) ribosylation and decrease in sympathetic tone [73].

Another research group studied the effect of ischemic injury on the vascular level. It is known that ischemic injury causes an increase in the level of metalloproteinases that degrade the endothelium of cerebral vessels. AG prevented the rise in metalloproteinases (MMP2, MMP9) in murine brain endothelial cell cultures exposed to oxygen–glucose deprivation–reperfusion injury [74].

AG exhibited cardioprotective properties by modulating calcium metabolism in isolated ventricular myocytes from Sprague Dawley rats [75,76]. Its potential was further assessed in a chronic cardiotoxicity model induced by doxorubicin in male Wistar rats. Doxorubicin increased the stimulation threshold of the papillary muscle while reducing its contractile strength; these effects were completely prevented by AG administration, which caused a decrease in left ventricular papillary muscle contraction. Moreover, AG protected against the doxorubicin-induced impairment of ventricular contraction (QRS) and repolarization (QT interval), as well as disturbances in sinoatrial node pacemaker activity and atrial impulse conduction, although it did not affect alterations in heart rate (RR interval). Additionally, AG reduced inflammation and cardiac cell injury produced by doxorubicin and enhanced the plasma’s antioxidant capacity. Importantly, AG prevented mortality, with 0% deaths observed in the group treated with both doxorubicin and AG, compared to 40% mortality in the group receiving doxorubicin alone [77].

In another study using a model of cardiotoxicity induced by isoproterenol in rats, AG improved cardiac contractility, decreased HR, and provided protective effects on both ventricular contraction and relaxation, as well as atrial contraction. AG’s cardioprotective actions against isoproterenol toxicity were attributed to its antioxidant properties and its ability to stabilize calcium balance. The researchers suggested that these benefits were mediated by an activation of the Nrf2 signaling pathway and a prevention of calcium overload in cardiac tissue [78].

AG has anti-ischemic properties on heart and vascular injury and exhibits cardioprotective effects on cardiotoxicity induced by doxorubicin and isoproterenol.

### 3.6. The Effects on Endothelial Dysfunction and Atherosclerosis of AG

AG has also been shown to offer protection against the progression of atherosclerosis and endothelial dysfunction. In studies using male New Zealand white rabbits on a 0.5% cholesterol-enriched diet (HED), the administration of AG at a dose of 10 mg/kg resulted in a decrease in total cholesterol and low-density lipoprotein (LDL) cholesterol, while increasing high-density lipoprotein (HDL) cholesterol. AG effectively inhibited the advancement of atherosclerosis in these rabbits, as seen by a smaller atherosclerotic lesion area (3.9 ± 0.27 μm^2^/10 μm compared to 7.7 ± 0.63 μm^2^/10 μm in control), fewer foam cells, and a lower intima/media ratio in the isolated aorta. Furthermore, AG ameliorated endothelial dysfunction caused by atherosclerosis, as indicated by lower malondialdehyde (a marker of oxidative stress), decreased lactate dehydrogenase (a marker of cellular injury), and reduced NOx levels [79]. In a separate study involving apoE-knockout mice, which spontaneously develop dyslipidemia and arterial lesions, the administration of AG produced anti-atherosclerotic effects. Treatment with AG reduced the development of aortic atherosclerotic lesions, improved lesion composition, elevated HDL levels, and upregulated the expression of genes involved in fatty acid metabolism [80].

It is well known that diabetes influences endothelial functions. In a rat model of diabetes induced by streptozotocin, endothelium-dependent relaxation was impaired. A treatment with AG improved acetylcholine-induced relaxation and also resulted in lower blood glucose levels [81].

Furthermore, in a study using human microvascular endothelial cell cultures where dysfunction was triggered by palmitate, AG improved mitochondrial function—an important factor in atherosclerosis—by activating the AMPK/PI3K/Akt/eNOS pathway [82].

In another experimental animal model of endothelial dysfunction induced by nicotine administration, AG markedly improved the lipid profile, decreased lipid peroxidation, enhanced antioxidant capacity (as shown by lower MDA and higher SOD and GDH), restored NO levels, and suppressed both the expression of the pro-inflammatory transcription factor NF-B and the serum concentration of VCAM-1 [83].

AG also showed protective effects against endothelial dysfunction in a rat model of endotoxemia induced by LPS injection [84].

Jo and collaborators [85] analyzed plasma AG levels in 322 individuals with metabolic syndrome. Their findings revealed that the average plasma AG concentration was lower in the participants with metabolic syndrome (79.42 ng/mL) compared to those without it (82.44 ng/mL). These results suggest that AG may represent a biomarker or a therapeutic target in metabolic syndrome [85].

Obesity is well recognized as a cardiovascular risk factor, contributing to both hypertension and endothelial dysfunction. Recent studies using rodent models of obesity have revealed that cardiac AG levels are significantly reduced in these conditions, as determined using high-performance liquid chromatography. Notably, lower AG concentrations were found to be negatively correlated with obesity and hypertension, as well as cardiac remodeling and dysfunction [86].

AG attenuated endothelial dysfunction and had anti-atherosclerotic effects (lower total cholesterol and LDL-cholesterol, increased HDL) and antioxidant effects.

### 3.7. The Effects of AG at the Cardiomyocytes Level

It has been found that α2R and I1R are present on the ventricular myocyte sarcolemma of Sprague Dawley and Wistar rats. As an agonist of both receptors, AG exhibited dual effects, depending on the dose. At a low dose (100 μM), AG activated α2R and decreased intracellular calcium via the PI3K/Akt/eNOS signaling pathway and increased calcium uptake in the endothelial reticulum via SERCA. At a high dose (15 mM), AG activated I1R and increased intracellular calcium via the PC-PLC/DAG/PKC/eNOS signaling pathway [87]. Also, it has been demonstrated that AG modulated calcium homeostasis by arachidonic acid-regulated calcium permeable (ARC) channels and store-operated calcium entry (SOC) in hibernating ground squirrels [88]. AG reduced catecholaminergic stress induced through isoprotenerol activation of βR in the left ventricular cardiomyocytes of Wistar rats through the serine–threonine protein phosphatase (STPP) dephosphorylation of VGCC in the I1-mediated signal [89]. Even though AG retained its effects on intracellular calcium modulation in the spontaneous hypertensive rat (SHR) model, research has demonstrated a disruption in signaling through α2R [90] and I1R [91] in this experimental model.

AG promoted the migration of murine brain endothelial cells (bEnd.3 cells) via the activation of VEGF/VEGFR2 and the consequential PI3K/Akt/eNOS/NO/ICAM-1 signaling pathways [92].

### 3.8. Strengths, Limitations, and Future Directions

This review is the first review that aims to systematize the results obtained from preclinical studies that evaluate the cardiovascular effects of AG. It is a critical, concise, and comprehensive systematic review, and it opens up future prospects for research on AG in human subjects.

The limitations of this review are the exclusion of articles without a full text available and with a full text in a language other than English, and the use of only three databases.

## 4. Conclusions

**Figure 5:**
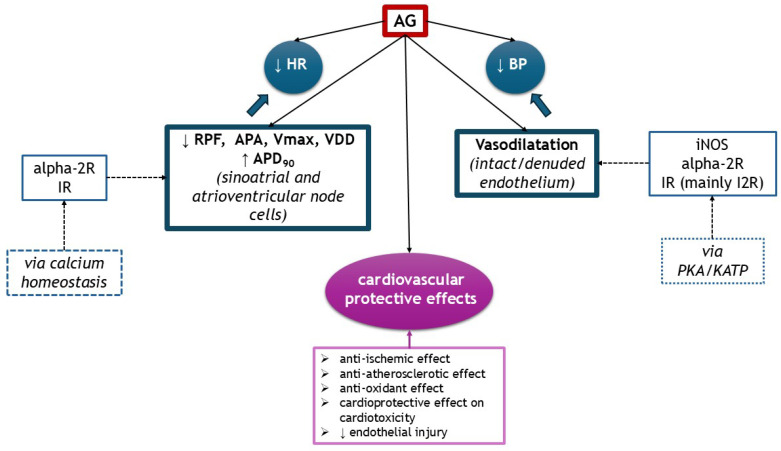
The cardiovascular effects of AG (↑ increased, ↓ decreased). alpha-2R, alpha-2 adrenergic receptors; APA, action potential amplitude; APD_90_, action potential duration at 90% of repolarization; BP, blood pressure; IR, imidazoline receptors; KATP, ATP-sensitive potassium channel; HR, heart rate; RPF, rate of pacemaker firing; PKA, protein kinase A; Vmax, maximum upstroke velocity of phase 0 depolarization; VDD, velocity of diastolic (phase 4) depolarization.

AG exhibited multiple cardiovascular effects, depending on the dosage, substrate, and route of administration. It acts on several receptors, such as IR and alpha-2R, but also on NOS isoforms. It exerts hypotensive effects, centrally or peripherally by vasodilatation. It influences inotropism or chronotropism through modulating calcium homeostasis in sinoatrial and atrioventricular cells or ventricular cardiomyocytes. AG has anti-ischemic properties on heart and endothelial injury and exhibits cardioprotective effects on cardiotoxicity induced by doxorubicin and isoproterenol. It also attenuated endothelial dysfunction through its anti-atherosclerotic (lower total cholesterol and LDL-cholesterol, increases HDL-cholesterol) and antioxidant properties. The cardiovascular effects of AG are illustrated in Figure 5.

## Figures and Tables

**Table 1:** Cardiovascular effects of central and peripheral administration of AG (↑ increased, ↓ decreased) BP, blood pressure; icv, intracerebroventricularly; iv, intravenous; HR, heart rate; SHR, spontaneous hypertensive rat.

Routes of Administration		HR	BP	Dose	Model of Animal Experiment	Studies
Centrally	Icv	-	↑	10–1000 nmol/5 μL	Anesthetized SHR	[39]
		-	↑	10–1000 nmol/5 μL	Anesthetized SHR	[40]
	Into the fourth ventricle	↑	-	10–1000 nmol/5 μL	Anesthetized SHR	[39]
		↑	-	10–1000 nmol/5 μL	Anesthetized SHR	[40]
		↑	-	30–300 μg/kg	SHR	[24]
			↑	>1000 μg/kg + adverse effects	SHR	[24]
Peripherally	Iv	↓	↓	0.01–100 mg/kg	Anesthetized > pithed SHR	[39]
		↓	↓	0.01–100 mg/kg	Anesthetized and pithed SHR	[24]

## Data Availability

No new data were created or analyzed in this study.

## Supplementary Materials

The following supporting information can be downloaded at: https://www.mdpi.com/article/10.3390/medsci13040255/s1, Table S1: Cardiovascular effects of AG.

## Abbreviations

The following abbreviations are used in this manuscript:

AG, agmatine; NO, nitric oxide; I1R, imidazoline 1 receptor; I2R, imidazoline 2 receptor; NOS, nitric oxide synthase; nNOS, neuronal nitric oxide synthase; iNOS, inducible nitric oxide synthase; eNOS, endothelial nitric oxide synthase; NMDAR, N-methyl-D-aspartate receptors; CNS, central nervous system; RVLM, rostral ventrolateral medulla; CDS, clonidine-displacing substance; iv, intravenous; BP, blood pressure; HR, heart rate; NA, norepinephrine; SHR, spontaneous hypertensive rat; icv, intracerebroventricularly; LDL, low-density lipoprotein; HDL, high-density lipoprotein.
