# Supplementary material for: Exploring the Cardiovascular Impacts of Agmatine: A Systematic Review
Source: Med Sci (Basel). 2025 Oct 31;13(4):255. doi: 10.3390/medsci13040255 (PMC12641643; doi:10.3390/medsci13040255)
Supplement: Supplementary file 1 [file medsci-13-00255-s001.zip › medsci-3930309-supplementary.pdf]

Exploring the Cardiovascular Impacts of Agmatine: A Systematic Review

Oana-Mădălina Manole, Gabriela Rusu-Zota, Amin Bazyani and Viviana Onofrei

Table S1. Cardiovascular effects of AG.

Effect	Mechanism involved	Sample	Study
Cardiac effects			
↓ HR	IR	male and female cross-bred conscious rabbits	[34-35]
did not alter HR		Sprague-Dawley rats (intrarenal or intracerebroventricular administration)	[38]
↓automaticity of pacemaker cells in SA node (↓VDD, RPF), ↓ transmembrane potentials of pacemaker cells in the SA node (↓Vmax,↑APD_{90})	Via IR and α2R by reducing the calcium influx and potassium efflux	SA node from the right atrium of rabbits	[59]
↓ early and delayed afterdepolarization induced by isoproterenol	Related to calcium influx mediated by IR and α2R	guinea pig papillary muscles	[61]
↓HR		anesthetized more than pithed SHR (intravenous administration)	[39]
↑HR	due to the nuleus tractus solitarius (administration ICV had no effect)	anethezied SHR (into the fourth ventricle administration)	[39]
Improves coronary flow and mechanical left ventricular performance; reduces cell damage (ip)	Via I2R and NO	Isolated ischemic-reperfused hearts from male Wistar rats	[72]
↓spontaneous activity of AV node cells (↓ APA, Vmax, VDD and RSF; ↑ APD_{90})	Mediated by calcium influx and potassium efflux via IR and/or α2R	AV node cells from rabbits	[60]
did not alter inotropy	via more postsynaptic αR rather than IR	left atria from Male Wistar rats	[62]
↓automaticity and transmembrane potentials	Blocking calcium influx via IR or alpha-2R	human atrial fibers from the apex of the right atrium appendage	[71]
Inhibited the carotid baroreflex	via IR and α2R	Isolated carotid sinus from Sprague-Dawley rats	[56]

Improve mechanical left ventricular performance and myocardial recovery (ip low doses pre- and post- and high doses only pre-ischemia)	Inhibition of the poly(ADP)ribosylation and a decrease in sympathetic tone	Isolated ischemic-reperfused hearts from male Wistar rats	[73]
↓ HR<br>↓ left ventricular blood pressure<br>↓ the first derivative of LVP (LP dp/dt)<br>↓ cardiac index (=CO/100g bw)<br>↓ cardiac output	mainly by I1, with the participation of I2 and α2R	Anesthetized Dahl salt-sensitive rat (DS) (hypertensive by high-sodium diet) vs Dahl salt-resistant rat (DR) normotensive	[57]
cardioprotective effects	↓ L-type calcium channel current slows down the recovery<br>of the calcium channel from inactivation	isolated ventricular myocytes from adult male Sprague-Dawley rat	[75]
cardioprotective effects	Inhibited calcium influx by inhibiting the voltage-dependent calcium channels and the release of calcium from SR	isolated Sprague-Dawley rat ventricular myocytes	[76]
↓HR (more in anesthetized than pithed SHR)	probably central mechanism -may modulate NA release in the same way as clonidine via IR and involves a reduction in sympathetic tone	Pithed SHR rats and anesthetized SHR (intravenous administration)	[40]
↑HR		Anesthetized SHR (into the fourth ventricle)	[40]
↓rate of pacemaker fires and APD	did not involve α2R	SA node pacemakers cells from Male New Zealand white rabbits	[47]
↑HR	By blocking glutaminergic facilitatory interneurons that activate the vagus cranial nerve	conscious SHR (lower doses, into the fourth ventricle administration)	[24]
↓HR<br>↓BP<br>Blocked the baroreflex induced by electrical stimulation of the left aortic nerve<br>abolition of L-glutamate-induced pressor action	IR or adrenoceptors receptors	anesthetized adult male Sprague-Dawley rats (into RVLM administration)	[55]

inotropic effect (↑ transient additional stimulatory contractions)	via prejunctional α2R located on sympathetic nerve terminals	frog isolated ventricular strips	[63]
↓MMP9	Dependent on NO	Murine brain endothelial cells and bEnd.3 cells exposed to oxygen-glucose deprivation reperfusion injury	[74]
↓MMP2	Independent of NO	Murine brain endothelial cells and bEnd.3 cells exposed to oxygen-glucose deprivation reperfusion injury	[74]
Positive inotropic and chronotropic effects	IR and adrenoceptors receptors	Isolated rat heart atria from Male Wistar rats	[64]
↓HR (sympatho-inhibition) produced by electrical sympathetic stimulation	involves α2 and I1	Pithed Male Wistar normotensive rats	[65]
↑contraction force at low stimulation frequencies	Activating SOC-channels	Right ventricle papillary muscle of ground squirrels	[70]
↓HR (sympatho-inhibition) produced by electrical sympathetic stimulation and NA (only in higher doses)	mediated by prejunctional α2R and I1R at the same extent (in SHR the inhibitory functions of α2R is altered)<br>-lowest dose of AG – prejunctional α2R (electrical stimulation)<br>-highest dose of AG – β1 blockade postjunctional (electrical and NA stimulation)	Pithed male SHR and pithed male Wistar normotensive rats	[66]
↓HR (sympatho-inhibition)	via α2AR mainly, α2CR and I1R	male Wistar normotensive rats pithed	[67]
protective effect on doxorubicin cardiotoxicity (protective effect on ventricular contraction (QRS), ventricular repolarization activity (QT interval), not on RR interval (HR), PR interval (sinoatrial node pacemaker firing and conduction of SA node	-The cardiotoxicity of doxorubicin administration is due<br>to calcium ions overload in myocytes<br>-AG:<br>↑uptake of cytosolic calcium, ↓sarcoplasmic calcium leakage,↑activity of calcium pumps include	male Wistar rats	[77]

impoulse through atrium);<br>prevented death, cardiac inflammation, cardiac cell damage			
Improve contractile force, ↓HR, ECG parameters, and oxidative markers	by antioxidant effects and controlling the hemostasis of calcium in myocytes (could be linked to the activation of the Nrf2 signaling pathway and inhibition of calcium overload in cardiac tissue)	Cardiotoxicity induced in male Wistar rats by sc isoprotenerol (a non-selective β-adrenergic agonist) administration (85 mg/kg/day, two days)	[78]
Vascular effects			
Restore the contractile response in the endothelium-free aorta pretreated with endotoxin	inhibit iNOS	The thoracic aorta from Sprague Dawley rats	[41]
↓ systemic arterial pressure and systemic vascular resistance tachyphylaxis	Via IR	Anesthetized Sprague-Dawley rats in vivo	[33]
↓arterial pressure	Via inhibiting postganglionic sympathetic nerve output, probably within the ganglion and direct action on vascular smooth muscle	anesthezied, ventilated adult male Sprague Dawley rats (intravenous administration)	[37]
↑arterial pressure	can not be attributed to direct actions on vasomotor neurons of the RVL	anesthezied, ventilated adult male Sprague Dawley rats (intracisternal administration)	[37]
Did not alter BP (↑ sodium excretion)	possibly due to IR	Sprague-Dawley rats (intrarenal or intracerebroventricular)	[38]
inhibit the vasoconstriction	activating prejunctional α2R	tail artery from male Sprague Dawley rats	[43]
↓BP		anesthetized more than pithed SHR (iv or into the fourth ventricle administration)	[39]
↑BP	Involve projection fields of forebrain areas to the pressor centers within RVLM	anesthetized SHR (intracerebroventricular administration)	[39]
↓BP<br>vasodilatation	independent of the endothelium, NO, or cGMP	Rats and rabbits;<br>isolated thoracic aorta rings, precontracted with	[44]

		phenylephrine and noradrenaline;	
↑ pressure perfusion (vasoconstriction)	Mainly α2R	Femoral vascular bed	[45]
Byphasic, ↑ pressure perfusion - vasoconstriction (lower doses) and ↓ pressure perfusion - vassodilatation (higher doses)	α2R≥IR	Renal vascular bed	
↓ pressure perfusion - Vassodilatation	Mainly IR	Mesenteric vascular bed	
↓ total peripheral resistance index (TPRI=MAP/CI) vassodilatation	mainly by I1R, with the participation of I2R and α2R	anesthetized Dahl salt-sensitive rat (DS) (hypertensive by high-sodium diet) vs Dahl salt-resistant rat (DR) normotensive	[57]
↓BP (more in anesthetized than pithed SHR)	may modulate NA release in the same way that clonidine does via IR	pithed SHR and anesthetized SHR (iv administration)	[40]
↑BP		anesthetized SHR (into central lateral ventricle)	[40]
did not alter the contractility of intact or endothelium-denuded aortal rings		The thoracic aorta of rats	[40]
contribute to the Ach relaxation responses	By ↓ blood glucose level and antioxidant effect	The thoracic aorta from adult male and female Wistar rats with Streptozotocin-diabetes induced	[81]
did not produce vasoconstriction		thoracic aorta and ear vein of male New Zealand white rabbit	[47]
vasodilatation endothelium-intact rings	stimulating NOS by an I1R-independent mechanism	endothelium-denuded and intact rat aortic rings isolated from male Sprague–Dawley rats	[48]
↓BP (greater and long-lasting if is coadministered with an NOs inhibitor)	may amplify the baroreceptor reflex by affecting the central sympathetic outflow	thoracic aorta from Wistar-Kyoto male rats	[54]
reversed the vasoconstriction induced by phenylephrine		thoracic aorta from Wistar-Kyoto male rats	[54]

attenuated relaxant responses to acetylcholine		thoracic aorta from Wistar-Kyoto male rats	[54]
did not affect BP	not only by α2R	conscious SHR (lowest doses, into the fourth ventricle, or cumulative intracisternal administration after clonidine or rilmenidine injection)	[24]
long-lasting ↑BP	not only by α2R	conscious SHR<br>(higher doses into fourth ventricle)	[24]
Biphasic effect – firstly inhibited, then enhanced – vasoconstriction induced by sympathetic nerve stimulation	prejunctional adrenoceptors-independent mechanism	male New Zealand white rabbit isolated saphenous artery without endothelium	[49]
vascular relaxation	via activation of proteinkinase B/Aktt/eNOS/GMP/ small conductance Ca-activated K channels or ATP-sensitive K channels	thoracic aorta of male Sprague Dawley rats (pretreated with phenylephrine)	[50]
↓SBP in SHR	Via I2R	Male SHR and Wistar-Kyoto rats	[52]
Vascular relaxation	Via I1R<br>PKA/KATP	Isolated aortic rings from SHR	[52]
	via α2AR and NO	isolated mesenteric arteriole from male Sprague-Dawley rats and Dahl salt-sensitive rats	[51]
↓diastolic BP	on the vascular sympathetic discharge via a prejunctional mechanism	pithed male Wistar normotensive rats	[58]
↓ mean arterial pressure	mainly by I1R, with the participation of I2R and α2R	anesthetized Dahl salt-sensitive rat (DS) (hypertensive by high-sodium diet) vs Dahl salt-resistant rat (DR) normotensive	[57]
↓SBP in SHR<br>Attenuated the tonic contraction of SHR aortic rings induced by phenylephrine and KCl		SHR and aortic rings isolated from SHR	[52]
Induced relaxation of aortic rings from rats	by opening ATP-sensitive potassium channels via peripheral I2R	isolated aorta rings from male Wistar rats	[53]

precontracted with phenylephrine and KCl			
Suppresses peripheral sympathetic tone and attenuated ganglionic transmission in the peripheral sympathetic nervous system	-reduced the EFS-evoked NA release via IR2 activation and by inhibition of ICa-N, with a decrease of intracellular calcium concentration in the peripheral nerve terminals.<br>-suppressed AP firing in SCG neurons	Denuded mesenteric aorta from male Sprague Dawley rats<br><br>AG suppressed the peripheral sympathetic tone by inhibiting N-type calcium channels via IR2 activation (Kim et al., 2016).	[68]
Inhibited the vasodepressor sensory CGRPergic outflow	mediated by a prejunctional activation of I1R on perivascular sensory nerves	Pithed male Wistar rats	[69]
↓progression of atherosclerosis	↓total cholesterol and LDL-cholesterol low-density lipoprotein cholesterol<br>↑HDL-cholesterol high-density lipoprotein cholesterol;<br>↓MDA, LDH, NOx	adult male New Zealand white rabbits<br>fed with 0.5% cholesterol-enriched diet	[79]
Protective effects against nicotine-induced endothelial dysfunction	↑ SOD, GDH<br>↓MDA<br>↑NOx<br>↓TC, TG, LDL-c<br>↑HDL<br>↓NF-B, VCAM-1	male New Zealand White rabbits with vascular endothelial dysfunction induced by nicotine	[83]
protective effects against endothelial dysfunction	↓LDH, CRP, CK-MB, MDA, NOx, iNOS, mortality<br>↑GSH, SOD	Male Sprague Dawley rats model of endotoxemia induced by LPS injection	[79]
anti-atherogenic action	↓ atherosclerotic lesions in the aorta<br>favorably modified lesion composition<br>↑ HDL levels<br>↑ expression of factors involved in fatty acid metabolism.	apoE-knockout mice	[80]
↑cell viability<br>↓LDH release<br>↑NO production<br>↑EI-1, ET-A<br>↓TC and TG accumulation<br>↑ATP, ↓mitochondrial ROS, ↑OCR	Via AMPK/PI3K/Akt/eNOS pathway	Endothelial dysfunction induced by palmitate (a type of free fatty acid) in human microvascular endothelial cell culture	[82]

(mitochondrial dysfunction, a contributor to atherosclerosis, was ameliorated by AG)			
Low level of AG		Male Wistar rats	[86]

APA, amplitude of action potential; Vmax, maximal rate of depolarization; VDD, velocity of diastolic (phase 4) depolarization; RPF, rate of pacemaker firing; APD_{90}, 90% duration of action potential
